# Crystal structure of a water oxidation catalyst solvate with composition (NH_4_)_2_[Fe^IV^(*L*-6H)]·3CH_3_COOH (*L* = clathrochelate ligand)

**DOI:** 10.1107/S2056989023010514

**Published:** 2024-01-01

**Authors:** Maksym O. Plutenko, Sergii I. Shylin, Sergiu Shova, Aleksander V. Blinder, Igor O. Fritsky

**Affiliations:** a Department of Chemistry, Taras Shevchenko National University of Kyiv, 01601 Kyiv, Ukraine; b Department of Chemistry - Ångström Laboratory, Uppsala University, 75335, Uppsala, Sweden; c "Petru Poni" Institute of Macromolecular Chemistry, Department of Inorganic, Polymers, 700487 Iasi, Romania; d Innovation Development Center ABN, Pirogov Str. 2/37, 01030 Kyiv, Ukraine; Vienna University of Technology, Austria

**Keywords:** clathrochelate, cage complex, high-valent iron, template synthesis, crystal structure

## Abstract

The synthetic availability of mol­ecular water oxidation catalysts containing high-valent ions of 3*d* metals in the active site is a prerequisite to enabling photo- and electrochemical water splitting on a large scale. Herein, the synthesis and crystal structure of di­ammonium {μ-1,3,4,7,8,10,12,13,16,17,19,22-dodeca­aza­tetra­cyclo­[8.8.4.1^3,17^.1^8,12^]tetra­cosane-5,6,14,15,20,21-hexa­onato}ferrate(IV) acetic acid tris­olvate, (NH_4_)_2_[Fe^IV^(C_12_H_12_N_12_O_6_)]·3CH_3_COOH or (NH_4_)_2_[Fe^IV^(*L*–6H)]·3CH_3_COOH is reported. The Fe^IV^ ion is encapsulated by the macropolycyclic ligand, which can be described as a dodeca-aza-quadricyclic cage with two capping tri­aza­cyclo­hexane fragments making three five- and six six-membered alternating chelate rings with the central Fe^IV^ ion. The local coord­ination environment of Fe^IV^ is formed by six deprotonated hydrazide nitro­gen atoms, which stabilize the unusual oxidation state. The Fe^IV^ ion lies on a twofold rotation axis (multiplicity 4, Wyckoff letter *e*) of the space group *C*2/*c*. Its coordination geometry is inter­mediate between a trigonal prism (distortion angle φ = 0°) and an anti­prism (φ = 60°) with φ = 31.1°. The Fe—N bond lengths lie in the range 1.9376 (13)–1.9617 (13) Å, as expected for tetra­valent iron. Structure analysis revealed that three acetic acid mol­ecules additionally co-crystallize per one iron(IV) complex, and one of them is positionally disordered over four positions. In the crystal structure, the ammonium cations, complex dianions and acetic acid mol­ecules are inter­connected by an intricate system of hydrogen bonds, mainly *via* the oxamide oxygen atoms acting as acceptors.

## Abstract 2

The coordination polyhedron of the complex [Fe^IV^(C_12_H_12_N_12_O_6_)]^2−^ or [Fe^IV^(*L*–6H)]^2−^ anions exhibits a shape inter­mediate between trigonal-prismatic and anti­prismatic.

## 1. Chemical context

The design of robust and efficient water oxidation catalysts based on 3*d* metals requires a rational approach that considers both their redox properties and crystal structure (Blakemore *et al.*, 2015 ▸). The intrinsic lability of the *M—L* bonds (*M* = central 3*d* metal cation, *L* = ligand) in aqueous solution is one of the main design challenges (Gil-Sepulcre & Llobet, 2022 ▸). In addition, the ligand in the catalyst has to be simple and oxidatively robust, otherwise it will be oxidized in the course of the catalysis (Boniolo *et al.*, 2022 ▸). Efficient chemical (Shylin *et al.*, 2019*a* ▸) and photochemical (Shylin *et al.*, 2019*b* ▸) water splitting using a clathrochelate complex Na_2_[Fe^IV^(*L*–6H)] as a catalyst has recently been reported. The relatively high reaction rate and turnover number have been attributed to the exceptional stability of this cage compound bearing the Fe ion in the unusual oxidation state +IV. Clathrochelate complexes [Fe^IV^(*L*–6H)]^2−^ with various cations (hexa­methyl­ene­tetra­minium, Bu_4_N^+^, Ph_4_As^+^, [Ca(H_2_O)_2_]^2+^, Li^+^) have been obtained and characterized structurally and spectroscopically (Tomyn *et al.*, 2017 ▸; Plutenko *et al.*, 2023 ▸). The Fe^IV^ ion can be reduced to Fe^III^ or oxidized to Fe^V^, either chemically or electrochemically, but at ambient conditions it spontaneously returns to the Fe^IV^ state in air, showcasing the stability of the oxidation state +IV in this specific ligand environment. Related compounds with [Mn^IV^(*L*–6H)]^2−^ clathrochelate anions have also been described recently (Shylin *et al.*, 2021 ▸). 
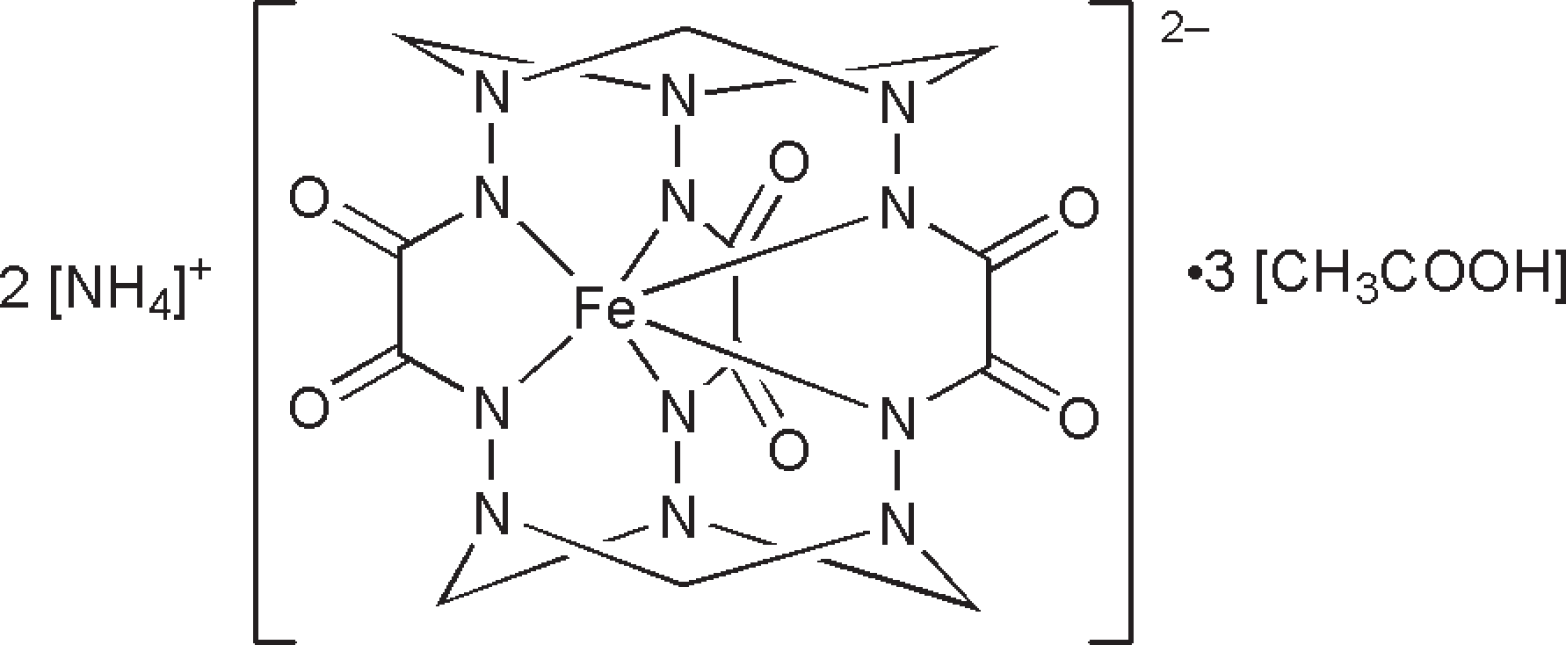


In this communication, we report on the template synthesis and crystal structure of the co-crystal compound (NH_4_)_2_[Fe^IV^(C_12_H_12_N_12_O_6_)]·3CH_3_COOH or (NH_4_)_2_[Fe^IV^(*L*–6H)]·3CH_3_COOH, which was obtained in an attempt to explore alternative crystallization strategies of cage compounds. We thus demonstrate that Fe^IV^ clathrochelates can be obtained in the form of single crystals under mild conditions.

## 2. Structural commentary

**Figure 1:**
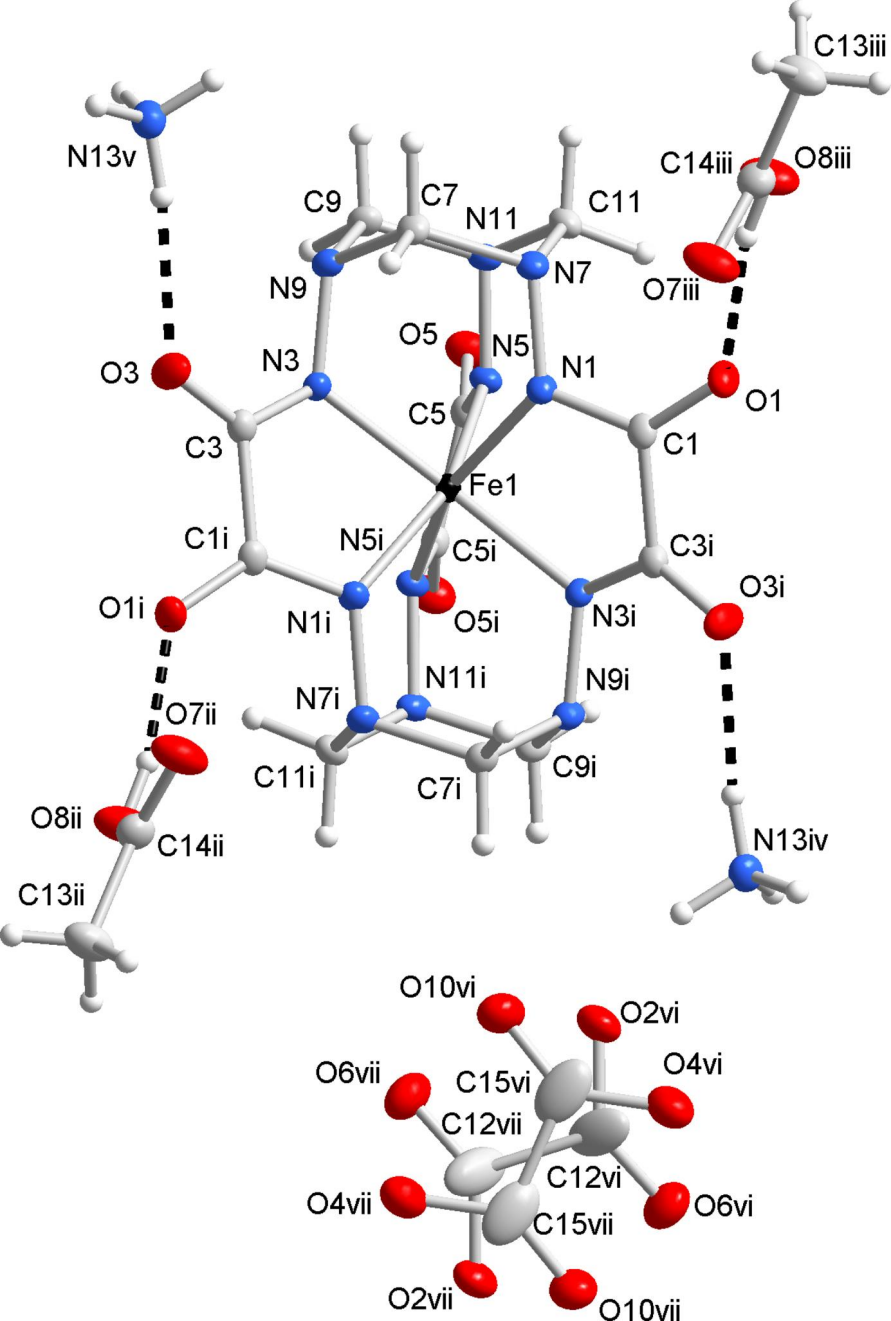
The mol­ecular moieties in the crystal structure of (NH_4_)_2_[Fe^IV^(l-6H)]·3CH_3_COOH with ellipsoids drawn at the 50% probability level. Hydrogen bonds are shown as dashed lines; H atoms of the disordered CH_3_COOH mol­ecule are omitted for clarity. [Symmetry codes: (i) 1 − *x*, *y*, 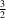 − *z*; (ii) 1 − *x*, 1 − *y*, 2 − *z*; (iii) *x*, 1 − *y*, − 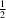 + *z*; (iv) − 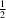 + *x*, − 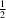 + *y*, *z*; (v) 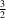 − *x*, 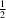 + *y*, 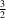 − *z*; (vi) − 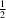 + *x*, 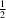 + *y*, *z*; (vii) 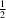 − *x*, 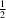 + *y*, 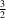 − *z*.]

The title compound consists of two ammonium cations, a clathrochelate dianion [Fe^IV^(*L*–6H)]^2−^, and three co-crystallized acetic acid mol­ecules per one formula unit (Fig. 1 ▸). The core of the macrocyclic ligand *L* is the hexa­hydrazide N-donor cage capped by two 1,3,5-tri­aza­cyclo­hexane fragments, thus featuring three five- and six six-membered chelate rings. All six hydrazide groups are deprotonated, and the formal charge of the ligand (*L*–6H) is 6–. The cage encapsulates the Fe ion in the oxidation state +IV, stabilized by the strong σ-donor capacity of the ligand (*L*–6H), as well as its ability to shield the ion from external factors. The shape of the coordination polyhedron [Fe^IV^N_6_] cannot be described as octa­hedral, which is typical for most ferrous and ferric complexes. It is rather inter­mediate between an ideal trigonal prism (*φ* = 0°) and an anti­prism (*φ* = 60°) with an average φ = 31.1°, calculated as a mean rotation of the N1—N3—N5 triangular base relative to N1^i^—N3^i^—N5^i^. It is within the range of 28.0–32.3° reported for other Fe^IV^ and Mn^IV^ clathrochelates (Tomyn *et al.*, 2017 ▸; Shylin *et al.*, 2021 ▸).

The Fe^IV^ ion lies on a special position of space group *C*2/*c* (twofold rotation axis, multiplicity 4, Wyckoff letter *e*), making half of the trigonal prism crystallographically independent. As such, the title compound is the first hexa­hydrazide complex with point group symmetry *C*_2_ for the complex anion, while all previous clathrochelates have symmetry *C*_1_ (Tomyn *et al.*, 2017 ▸; Shylin *et al.*, 2021 ▸; Plutenko *et al.*, 2023 ▸). The Fe—N bond lengths in (NH_4_)_2_[Fe^IV^(*L*–6H)]·3CH_3_COOH are between 1.9376 (13) and 1.9617 (13) Å, which are close to those reported for related compounds bearing the [Fe^IV^(*L*–6H)]^2−^ complex anion [1.915 (5)–1.969 (3) Å; Tomyn *et al.*, 2017 ▸]. The apical bite angles N—Fe—N fall in the range 86.36 (5)–87.56 (5)°, and equatorial bite angles are 80.01 (5)–80.18 (7)° (Table 1 ▸). The height of the trigonal prism (*i.e.* the distance between the triangular bases) is 2.374 (3) Å, which is in the range 2.36–2.38 Å reported for other clathrochelates (Tomyn *et al.*, 2017 ▸; Shylin *et al.*, 2021 ▸).

The macropolycyclic ligand in (NH_4_)_2_[Fe^IV^(*L*–6H)]·3CH_3_COOH exhibits noticeable distortions, especially with respect to the oxamide moieties. While the hydrazide groups O1–C1–N1–N7 and O3–C3–N3–N9 remain virtually planar, the oxamide moieties are significantly bent with noticeably large torsion angles around the C—C bonds. The O1—C1—C3^i^—O3^i^ and O5—C5—C5^i^–O5^i^ torsion angles are 20.15 (15) and 12.33 (16)°, respectively, with the larger torsion angle associated with O1 and O3 atoms involved in inter­molecular hydrogen bonding (see below). The five-membered chelate rings in the complex exhibit a non-symmetric twist conformation with N1—C1—C3^i^—N3^i^ and N5—C5—C5^i^—N5^i^ torsion angles of 20.11 (13) and 13.52 (15)°, respectively. The six-membered chelate rings have chair conformations with the Fe and C atoms deviating from the N_4_ mean plane, with corresponding dihedral angles in the range 35.45 (6)–36.38 (5)° and 59.50 (11)–60.62 (15)°, respectively.

One of three acetic acid mol­ecules is disordered, leading to four equivalent positions (Fig. 1 ▸). Specifically, the two C atoms of CH_3_COOH are disordered along the C—C bond – each can serve as either a methyl or a carboxyl C atom. They are additionally disordered between two positions each by means of the twofold symmetry axis. As such, occupancy factors of C and O atoms are 0.5 and 0.25, respectively.

## 3. Supra­molecular features

**Figure 2:**
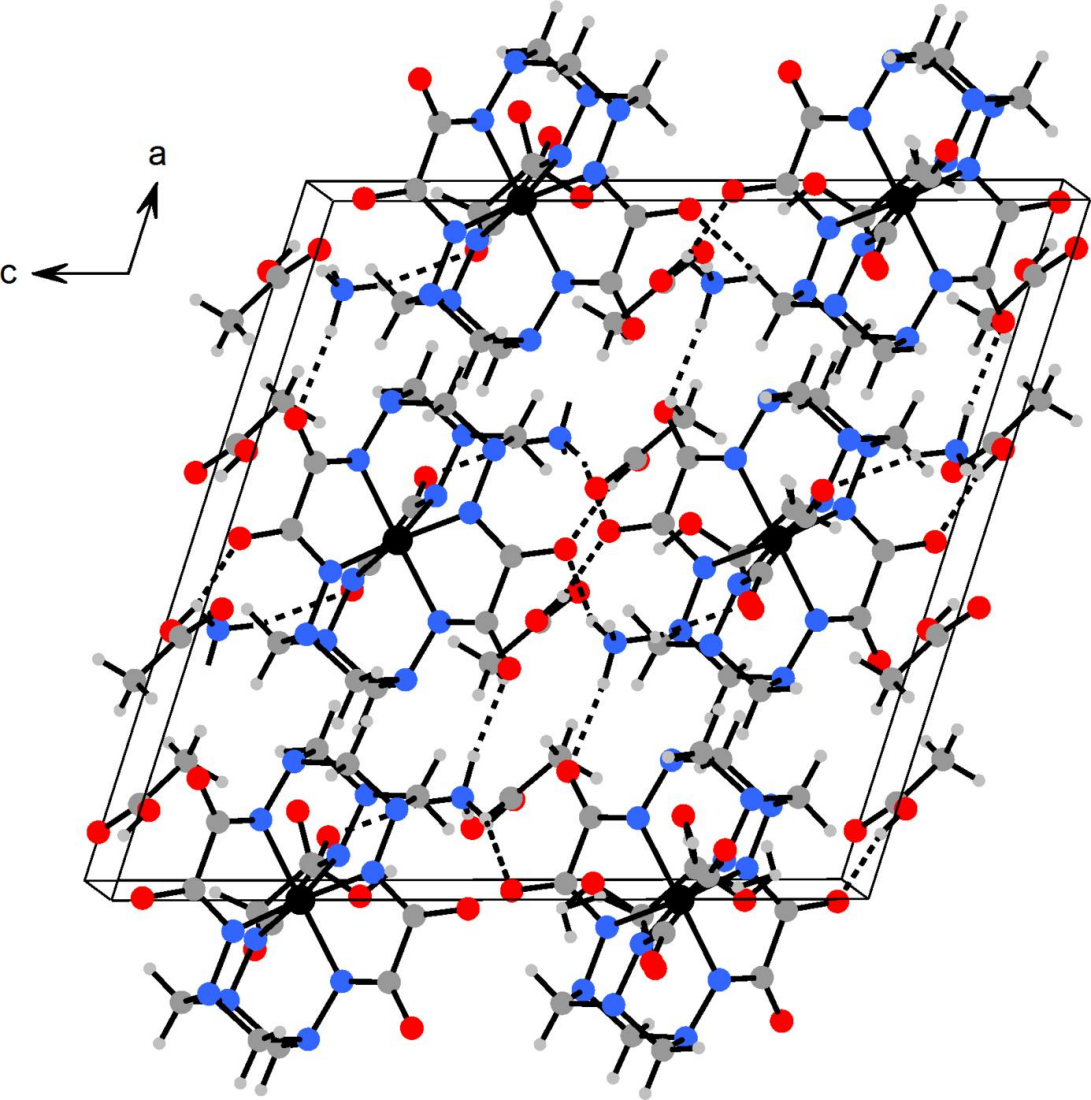
The crystal packing in (NH_4_)_2_[Fe^IV^(l-6H)]·3CH_3_COOH. Relevant hydrogen bonds are shown as dashed lines. The disordered CH_3_COOH mol­ecule is shown with only one possible orientation. Color code: Fe, black; N, blue; O, red; C, dark ray; H, gray; the unit-cell is outlined.

In the crystal structure of the title compound, the ammonium cations; complex anions and acetic acid mol­ecules are associated *via* an intricate set of O—H⋯O, N—H⋯O, N—H⋯N, and non-classical C—H⋯O hydrogen bonds (Table 2 ▸). Most of these contacts show angles far from linearity, indicating that they correspond to rather weak inter­actions. However, a few of them can be considered as significant inter­molecular contacts and are discussed in more detail. Each clathrochelate anion appears to be associated with two CH_3_COOH co-crystallized mol­ecules and four NH_4_^+^ cations, thus employing all six oxamide O atoms as acceptors for hydrogen bonding. The oxamide ribs of the clathrochelate exhibit different binding modes. Specifically, the (O1,O3) ribs are bound to CH_3_COOH and NH_4_^+^ through the O8—H8⋯O1^i^ and N13—H13*E*⋯O3^iii^ contacts, while the (O5,O5′) ribs are bound to two NH_4_^+^ ions through the crystallographically equivalent N13—H13*F*⋯O5 contacts (Fig. 2 ▸). The latter contacts are somewhat weaker than the former (note their *D*⋯*A* distances and angles, Table 2 ▸), which creates higher distortion of the oxamide moieties O1–C1–C3^i^–O3^i^ in favor of virtually linear hydrogen bonds. The non-protonated O atom of CH_3_COOH serves as an acceptor for another NH_4_^+^ proton, making N13—H13*D*⋯O7^ii^ contacts. The fourth remaining proton of NH_4_^+^ is involved in binding the neighboring clathrochelate anion through the N13—H13*G*⋯O1^iv^ contact.

All in all, the NH_4_^+^ cations, isolated complex anions and co-crystallized CH_3_COOH are connected into a tri-periodic supra­molecular framework by means of hydrogen bonds, mainly *via* oxamide O atoms as proton acceptors, and NH_4_^+^ and CH_3_COOH as donor groups.

## 4. Database survey

A search of the Cambridge Structural Database (CSD version 5.43, update of November 2022; Groom *et al.*, 2016 ▸) for complexes with the central metal cation coordinated by six hydrazide ligands revealed nine structures, six of them containing the clathrochelate complex [Fe^IV^(*L*–6H)]^2−^. Two of these structures represent mononuclear complexes with Bu_4_N^+^ and Ph_4_As^+^ cations (Tomyn *et al.*, 2017 ▸), and four are coordination polymers in which Ca^2+^ (Tomyn *et al.*, 2017 ▸), Mn^2+^ (Xu *et al.*, 2020*a* ▸), or Cu^2+^ (2 structures; Xu *et al.*, 2020*b* ▸) cations are exo-coordinated to the vacant (*O*,*O*′) and/or (*O*,*N*) chelating units of the hexa­hydrazide ligand. To the best of our knowledge, there has been only one structure of the Fe^IV^ hexa­hydrazide complex reported after November 2022 (Plutenko *et al.*, 2023 ▸).

## 5. Synthesis and crystallization

A powder of (Bu_4_N)_2_[Fe^IV^(*L*–6H)] was obtained by a metal template synthesis as described previously (Tomyn *et al.*, 2017 ▸). Then, 0.5 mmol of (Bu_4_N)_2_[Fe^IV^(*L*–6H)] and 1 mmol of CH_3_COONH_4_ were dissolved in 10 ml of water, and 10 ml of glacial acetic acid was added to this mixture. The resulting mixture was evaporated under vacuum on a rotary evaporator to a volume of *ca* 10 ml and left in a closed flask. After two weeks, dark-green crystals of (NH_4_)_2_[Fe^IV^(*L*–6H)]·3CH_3_COOH suitable for the X-ray diffraction analysis were obtained. FTIR (in KBr pellet, cm^−1^): 3424 (O—H), 3184 (N—H), 2953 (C—H), 1636 (C=O, amide I).

## 6. Refinement

Crystal data, data collection and structure refinement details are summarized in Table 3 ▸. The H atoms attached to C were placed in fixed idealized positions using a riding model with *U*_iso_(H) = 1.2*U*_eq_(C) for methyl­ene and 1.5 for methyl groups. The non-disordered H atoms attached to N and O were located in difference-Fourier maps and their positional parameters were verified according to the hydrogen-bonding geometry. Occupancy factors of C and O atoms of the disordered CH_3_COOH mol­ecule were fixed to 0.5 and 0.25, respectively. The H atoms attached to disordered O atoms were placed in fixed positions with *U*_iso_(H) = 1.5*U*_eq_(O), and their coordinates were refined according to the riding model described above.

## Supplementary Material

Crystal structure: contains datablock(s) I. DOI: 10.1107/S2056989023010514/wm5703sup1.cif

Structure factors: contains datablock(s) I. DOI: 10.1107/S2056989023010514/wm5703Isup2.hkl

Supporting information file. DOI: 10.1107/S2056989023010514/wm5703Isup3.cdx

Supporting information file. DOI: 10.1107/S2056989023010514/wm5703Isup4.cdx

CCDC reference: 2312686

Additional supporting information:  crystallographic information; 3D view; checkCIF report

## Figures and Tables

**Table 1:** Selected geometric parameters (Å, °) for the coordination polyhedron [Fe^IV^N_6_]

Fe1—N1	1.9610 (13)	Fe1—N5	1.9376 (13)
Fe1—N3	1.9617 (13)		
			
N1—Fe1—N3	86.36 (5)	N1—Fe1—N3^i^	80.01 (5)
N1—Fe1—N5	87.15 (5)	N5—Fe1—N5^i^	80.18 (7)
N5—Fe1—N3	87.56 (5)		

Symmetry code: (i) 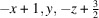 .

**Table 2:** Hydrogen-bond geometry (Å, °)

*D*—H⋯*A*	*D*—H	H⋯*A*	*D*⋯*A*	*D*—H⋯*A*
O2—H2⋯O7^ii^	0.84	1.94	2.763 (6)	165
O2—H12*B*⋯N1	1.00	2.66	3.338 (6)	125
O2—H12*A*⋯O5^iii^	1.17	2.58	3.356 (6)	122
O4—H4⋯O7^ii^	0.84	1.96	2.792 (7)	169
O8—H8⋯O1^iv^	0.81 (3)	1.80 (3)	2.6007 (17)	168 (3)
O10—H15*C*⋯O5^iii^	1.15	2.39	3.425 (6)	148
N13—H13*D*⋯O7^v^	0.94 (2)	1.99 (2)	2.913 (2)	166.4 (18)
N13—H13*E*⋯O3^vi^	0.91 (2)	2.00 (2)	2.9073 (19)	173.2 (19)
N13—H13*E*⋯N9^vi^	0.91 (2)	2.64 (2)	3.146 (2)	116.5 (16)
N13—H13*F*⋯O5	0.88 (2)	2.12 (2)	2.9606 (19)	160.7 (19)
N13—H13*F*⋯N11	0.88 (2)	2.63 (2)	3.252 (2)	128.3 (17)
N13—H13*G*⋯O1^vii^	0.87 (2)	2.09 (2)	2.9241 (19)	158.8 (19)
N13—H13*G*⋯O3^ii^	0.87 (2)	2.48 (2)	3.0698 (19)	125.0 (17)
C7—H3*A*⋯O5^viii^	0.99	2.47	3.4109 (19)	159
C7—H3*B*⋯O10	0.99	2.43	3.366 (6)	158
C9—H4*A*⋯O2^vi^	0.99	2.35	3.312 (7)	165
C9—H4*A*⋯O10^vi^	0.99	2.17	3.132 (5)	165
C11—H6*B*⋯O1^vii^	0.99	2.31	3.2606 (19)	160
C12—H12*B*⋯N1	0.98	2.66	3.575 (7)	155
C12—H12*A*⋯O5^iii^	0.98	2.58	3.448 (7)	147
C13—H13*B*⋯O5	0.90 (3)	2.67 (3)	3.480 (2)	151 (2)
C13—H13*C*⋯O8^ix^	0.91 (3)	2.63 (3)	3.416 (3)	146 (2)
C15—H15*C*⋯O5^iii^	0.98	2.39	3.245 (6)	146

Symmetry codes: (ii) 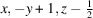 ; (iii) 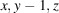 ; (iv) 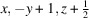 ; (v) 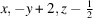 ; (vi) 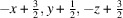 ; (vii) 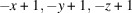 ; (viii) 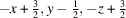 ; (ix) 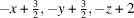 .

**Table 3:** Experimental details

Crystal data	
Chemical formula	(NH_4_)_2_[Fe(C_12_H_12_N_12_O_6_)]·3C_2_H_4_O_2_
*M*_r_	692.42
Crystal system, space group	Monoclinic, *C*2/*c*
Temperature (K)	120
*a*, *b*, *c* (Å)	15.5352 (2), 11.5178 (1), 16.0472 (2)
β (°)	107.616 (2)
*V* (Å^3^)	2736.70 (6)
*Z*	4
Radiation type	Mo *K*α
μ (mm^−1^)	0.64
Crystal size (mm)	0.45 × 0.20 × 0.13

Data collection	
Diffractometer	Rigaku SuperNova, Single source at offset, Eos
Absorption correction	Analytical [*CrysAlis PRO* (Rigaku OD, 2015 ▸) based on analytical numerical absorption correction using a multifaceted crystal model (Clark & Reid, 1995 ▸)]
*T*_min_, *T*_max_	0.888, 0.954
No. of measured, independent and observed [*I* > 2σ(*I*)] reflections	19869, 2431, 2345
*R*_int_	0.025
(sin θ/λ)_max_ (Å^−1^)	0.595

Refinement	
*R*[*F* ^2^ > 2σ(*F* ^2^)], *wR*(*F* ^2^), *S*	0.024, 0.064, 1.07
No. of reflections	2431
No. of parameters	265
H-atom treatment	H atoms treated by a mixture of independent and constrained refinement
Δρ_max_, Δρ_min_ (e Å^−3^)	0.31, −0.30

Computer programs: *CrysAlis PRO* (Rigaku OD, 2015 ▸), *SHELXT* (Sheldrick, 2015*a* ▸), *SHELXL* (Sheldrick, 2015*b* ▸), CrystaMaker (*CrystalMaker*, 2017 ▸), and *publCIF* (Westrip, 2010 ▸).

## supplementary crystallographic information

### Crystal data

**Table:**

(NH_4_)_2_[Fe(C_12_H_12_N_12_O_6_)]·3C_2_H_4_O_2_	*F*(000) = 1440
*M**_r_* = 692.42	*D*_x_ = 1.681 Mg m^−^^3^
Monoclinic, *C*2/*c*	Mo *K*α radiation, λ = 0.71073 Å
*a* = 15.5352 (2) Å	Cell parameters from 18947 reflections
*b* = 11.5178 (1) Å	θ = 3.8–34.6°
*c* = 16.0472 (2) Å	µ = 0.64 mm^−^^1^
β = 107.616 (2)°	*T* = 120 K
*V* = 2736.70 (6) Å^3^	Block, black
*Z* = 4	0.45 × 0.20 × 0.13 mm

### Data collection

**Table:**

Rigaku SuperNova, Single source at offset, Eos diffractometer	2431 independent reflections
Radiation source: micro-source	2345 reflections with *I* > 2σ(*I*)
Detector resolution: 16.0107 pixels mm^-1^	*R*_int_ = 0.025
φ scans and ω scans with κ offset	θ_max_ = 25.0°, θ_min_ = 3.2°
Absorption correction: analytical [CrysAlisPro (Rigaku OD, 2015) based on analytical numerical absorption correction using a multifaceted crystal model (Clark & Reid, 1995)]	*h* = −18→18
*T*_min_ = 0.888, *T*_max_ = 0.954	*k* = −13→13
19869 measured reflections	*l* = −19→19

### Refinement

**Table:**

Refinement on *F*^2^	0 restraints
Least-squares matrix: full	Hydrogen site location: mixed
*R*[*F*^2^ > 2σ(*F*^2^)] = 0.024	H atoms treated by a mixture of independent and constrained refinement
*wR*(*F*^2^) = 0.064	*w* = 1/[σ^2^(*F*_o_^2^) + (0.0287*P*)^2^ + 4.4687*P*] where *P* = (*F*_o_^2^ + 2*F*_c_^2^)/3
*S* = 1.07	(Δ/σ)_max_ < 0.001
2431 reflections	Δρ_max_ = 0.31 e Å^−^^3^
265 parameters	Δρ_min_ = −0.30 e Å^−^^3^

### Special details

**Table:**

Geometry. All esds (except the esd in the dihedral angle between two l.s. planes) are estimated using the full covariance matrix. The cell esds are taken into account individually in the estimation of esds in distances, angles and torsion angles; correlations between esds in cell parameters are only used when they are defined by crystal symmetry. An approximate (isotropic) treatment of cell esds is used for estimating esds involving l.s. planes.

### Fractional atomic coordinates and isotropic or equivalent isotropic displacement parameters (Å^2^)

**Table:**

	*x*	*y*	*z*	*U*_iso_*/*U*_eq_	Occ. (<1)
Fe1	0.500000	0.51456 (3)	0.750000	0.01084 (10)	
O1	0.49183 (7)	0.33179 (10)	0.53250 (7)	0.0185 (3)	
O3	0.67899 (8)	0.34064 (11)	0.94355 (8)	0.0230 (3)	
O5	0.58025 (8)	0.84207 (10)	0.72496 (8)	0.0219 (3)	
O7	0.58585 (9)	0.91495 (11)	1.02775 (9)	0.0309 (3)	
O8	0.62188 (9)	0.74720 (11)	0.97758 (10)	0.0306 (3)	
H8	0.5866 (19)	0.722 (2)	1.0013 (18)	0.054 (8)*	
N1	0.54198 (8)	0.41708 (11)	0.66990 (8)	0.0140 (3)	
N3	0.61583 (9)	0.47931 (11)	0.83762 (8)	0.0130 (3)	
N5	0.55986 (8)	0.64326 (11)	0.71280 (8)	0.0137 (3)	
N7	0.63190 (9)	0.42275 (12)	0.66460 (8)	0.0156 (3)	
N9	0.69873 (9)	0.48505 (11)	0.81754 (8)	0.0148 (3)	
N11	0.64693 (9)	0.63143 (12)	0.70168 (9)	0.0155 (3)	
N13	0.63652 (10)	0.84256 (13)	0.56441 (10)	0.0185 (3)	
H13D	0.6113 (14)	0.916 (2)	0.5469 (13)	0.028*	
H13E	0.6955 (16)	0.8442 (18)	0.5665 (13)	0.028*	
H13F	0.6296 (14)	0.8293 (18)	0.6159 (15)	0.028*	
H13G	0.6080 (14)	0.789 (2)	0.5275 (14)	0.028*	
C1	0.48051 (10)	0.37867 (13)	0.59867 (10)	0.0143 (3)	
C3	0.61482 (10)	0.39725 (13)	0.89673 (10)	0.0149 (3)	
C5	0.54073 (10)	0.75177 (14)	0.73270 (10)	0.0146 (3)	
C7	0.69757 (10)	0.39761 (14)	0.74949 (10)	0.0162 (3)	
H3A	0.758539	0.393247	0.742223	0.019*	
H3B	0.683704	0.320624	0.769698	0.019*	
C9	0.71270 (10)	0.60121 (14)	0.78697 (10)	0.0166 (3)	
H4A	0.774496	0.605943	0.781768	0.020*	
H4B	0.708119	0.658987	0.831114	0.020*	
C11	0.64715 (11)	0.54171 (14)	0.63704 (10)	0.0173 (3)	
H6A	0.705955	0.543798	0.624954	0.021*	
H6B	0.599543	0.560094	0.581832	0.021*	
C13	0.68764 (14)	0.91648 (18)	0.94181 (14)	0.0283 (4)	
H13A	0.7117 (17)	0.984 (2)	0.9689 (16)	0.042*	
H13B	0.6571 (16)	0.926 (2)	0.8850 (17)	0.042*	
H13C	0.7317 (17)	0.867 (2)	0.9373 (15)	0.042*	
C14	0.62703 (11)	0.86083 (15)	0.98682 (11)	0.0211 (4)	
O2	0.5695 (5)	0.1300 (5)	0.6914 (4)	0.0264 (14)	0.25
H2	0.564306	0.114115	0.639008	0.040*	0.25
O4	0.4878 (4)	0.1137 (5)	0.6462 (5)	0.0307 (15)	0.25
H4	0.523622	0.105568	0.616529	0.046*	0.25
O6	0.4189 (3)	0.1429 (5)	0.6588 (4)	0.0316 (12)	0.25
O10	0.6091 (4)	0.1341 (5)	0.7634 (4)	0.0303 (12)	0.25
C12	0.4935 (6)	0.1199 (6)	0.7018 (6)	0.039 (2)	0.5
H12A	0.520573	0.050427	0.684862	0.058*	0.25
H12B	0.520573	0.189367	0.684862	0.058*	0.25
H12C	0.428294	0.119897	0.672332	0.058*	0.25
C15	0.5341 (6)	0.1169 (5)	0.7245 (7)	0.041 (2)	0.5
H15C	0.571635	0.047433	0.741920	0.061*	0.25
H15B	0.571635	0.186372	0.741920	0.061*	0.25
H15A	0.506375	0.116903	0.660900	0.061*	0.25

### Atomic displacement parameters (Å^2^)

**Table:**

	*U*^11^	*U*^22^	*U*^33^	*U*^12^	*U*^13^	*U*^23^
Fe1	0.01181 (17)	0.01133 (17)	0.00910 (16)	0.000	0.00276 (12)	0.000
O1	0.0187 (6)	0.0216 (6)	0.0146 (6)	0.0041 (5)	0.0039 (4)	−0.0050 (5)
O3	0.0175 (6)	0.0266 (6)	0.0234 (6)	0.0029 (5)	0.0039 (5)	0.0125 (5)
O5	0.0253 (6)	0.0152 (6)	0.0282 (6)	−0.0036 (5)	0.0126 (5)	0.0001 (5)
O7	0.0368 (8)	0.0237 (7)	0.0423 (8)	−0.0063 (6)	0.0269 (7)	−0.0046 (6)
O8	0.0316 (7)	0.0218 (7)	0.0475 (8)	−0.0062 (6)	0.0254 (7)	−0.0035 (6)
N1	0.0126 (6)	0.0156 (7)	0.0138 (6)	0.0024 (5)	0.0038 (5)	−0.0008 (5)
N3	0.0114 (6)	0.0151 (6)	0.0120 (6)	−0.0015 (5)	0.0027 (5)	0.0009 (5)
N5	0.0130 (6)	0.0151 (7)	0.0149 (6)	0.0011 (5)	0.0067 (5)	0.0023 (5)
N7	0.0122 (6)	0.0197 (7)	0.0156 (7)	0.0035 (5)	0.0050 (5)	0.0006 (5)
N9	0.0117 (6)	0.0172 (7)	0.0153 (7)	−0.0006 (5)	0.0039 (5)	0.0019 (5)
N11	0.0132 (6)	0.0181 (7)	0.0170 (7)	0.0015 (5)	0.0074 (5)	0.0035 (5)
N13	0.0203 (8)	0.0174 (8)	0.0173 (7)	−0.0037 (6)	0.0048 (6)	−0.0026 (6)
C1	0.0174 (8)	0.0120 (7)	0.0128 (7)	0.0034 (6)	0.0033 (6)	0.0011 (6)
C3	0.0163 (8)	0.0149 (8)	0.0126 (7)	−0.0018 (6)	0.0033 (6)	−0.0007 (6)
C5	0.0160 (8)	0.0151 (8)	0.0115 (7)	−0.0003 (6)	0.0024 (6)	0.0012 (6)
C7	0.0143 (8)	0.0182 (8)	0.0159 (8)	0.0039 (6)	0.0041 (6)	0.0019 (6)
C9	0.0138 (7)	0.0178 (8)	0.0182 (8)	−0.0020 (6)	0.0049 (6)	0.0029 (6)
C11	0.0166 (8)	0.0221 (8)	0.0150 (8)	0.0036 (6)	0.0075 (6)	0.0031 (6)
C13	0.0318 (10)	0.0263 (10)	0.0330 (11)	−0.0074 (8)	0.0193 (9)	−0.0042 (8)
C14	0.0186 (8)	0.0230 (9)	0.0217 (9)	−0.0042 (7)	0.0058 (7)	−0.0019 (7)
O2	0.027 (4)	0.030 (3)	0.026 (4)	0.001 (2)	0.015 (3)	0.004 (2)
O4	0.031 (4)	0.035 (3)	0.030 (4)	0.005 (2)	0.015 (3)	0.004 (3)
O6	0.024 (3)	0.034 (3)	0.033 (3)	0.002 (2)	0.001 (3)	0.006 (2)
O10	0.024 (3)	0.035 (3)	0.033 (3)	−0.005 (2)	0.010 (3)	−0.003 (2)
C12	0.029 (4)	0.029 (3)	0.056 (7)	0.001 (3)	0.009 (4)	0.026 (3)
C15	0.041 (5)	0.015 (2)	0.053 (6)	−0.001 (3)	−0.005 (5)	0.007 (2)

### Geometric parameters (Å, º)

**Table:**

Fe1—N1	1.9610 (13)	C7—H3B	0.9900
Fe1—N1^i^	1.9610 (13)	C9—H4A	0.9900
Fe1—N3^i^	1.9617 (13)	C9—H4B	0.9900
Fe1—N3	1.9617 (13)	C11—H6A	0.9900
Fe1—N5	1.9376 (13)	C11—H6B	0.9900
Fe1—N5^i^	1.9376 (13)	C13—C14	1.493 (2)
O1—C1	1.2502 (19)	C13—H13A	0.92 (3)
O3—C3	1.2370 (19)	C13—H13B	0.90 (3)
O5—C5	1.2330 (19)	C13—H13C	0.91 (3)
O7—C14	1.218 (2)	O2—C12	1.246 (11)
O8—C14	1.317 (2)	O2—H2	0.8400
O8—H8	0.81 (3)	O2—H12A	1.1750
N1—C1	1.324 (2)	O2—H12B	1.0032
N1—N7	1.4266 (18)	O4—C15	1.244 (12)
N3—C3	1.342 (2)	O4—H4	0.8399
N3—N9	1.4205 (18)	O4—H15A	0.3140
N5—C5	1.345 (2)	O6—C12	1.186 (10)
N5—N11	1.4236 (17)	O6—H12C	0.3459
N7—C7	1.463 (2)	O10—C15	1.161 (10)
N7—C11	1.481 (2)	O10—H15C	1.1550
N9—C9	1.464 (2)	O10—H15B	0.8358
N9—C7	1.482 (2)	C12—C12^i^	1.50 (2)
N11—C11	1.465 (2)	C12—H12A	0.9800
N11—C9	1.481 (2)	C12—H12B	0.9800
N13—H13D	0.94 (2)	C12—H12C	0.9802
N13—H13E	0.91 (2)	C15—H15A	0.9798
N13—H13F	0.88 (2)	C15—C15^i^	1.52 (2)
N13—H13G	0.87 (2)	C15—H15C	0.9800
C1—C3^i^	1.520 (2)	C15—H15B	0.9800
C5—C5^i^	1.528 (3)	C15—H15A	0.9798
C7—H3A	0.9900		
			
N5^i^—Fe1—N1	157.78 (5)	N7—C11—H6A	108.8
N5—Fe1—N1^i^	157.78 (5)	N11—C11—H6B	108.8
N5^i^—Fe1—N1^i^	87.15 (5)	N7—C11—H6B	108.8
N1—Fe1—N1^i^	110.14 (8)	H6A—C11—H6B	107.7
N5—Fe1—N3^i^	111.05 (5)	C14—C13—H13A	111.3 (15)
N5^i^—Fe1—N3^i^	87.56 (5)	C14—C13—H13B	109.2 (15)
N1^i^—Fe1—N3^i^	86.36 (5)	H13A—C13—H13B	113 (2)
N1—Fe1—N3	86.36 (5)	C14—C13—H13C	111.9 (15)
N1^i^—Fe1—N3	80.01 (5)	H13A—C13—H13C	111 (2)
N1—Fe1—N5	87.15 (5)	H13B—C13—H13C	100 (2)
N5—Fe1—N3	87.56 (5)	O7—C14—O8	123.05 (16)
N1—Fe1—N3^i^	80.01 (5)	O7—C14—C13	123.51 (17)
N5^i^—Fe1—N3	111.05 (5)	O8—C14—C13	113.44 (15)
N5—Fe1—N5^i^	80.18 (7)	C12—O2—H2	107.9
N3^i^—Fe1—N3	156.12 (8)	C12—O2—H12A	47.6
C14—O8—H8	109 (2)	H2—O2—H12A	82.9
C1—N1—N7	115.28 (12)	C12—O2—H12B	50.2
C1—N1—Fe1	117.46 (10)	H2—O2—H12B	101.8
N7—N1—Fe1	122.60 (10)	H12A—O2—H12B	94.2
C3—N3—N9	113.47 (12)	C15—O4—H4	107.3
C3—N3—Fe1	116.60 (10)	C15—O4—H15A	28.6
N9—N3—Fe1	121.71 (9)	H4—O4—H15A	79.9
C5—N5—N11	113.90 (12)	C12—O6—H12C	46.5
C5—N5—Fe1	118.46 (10)	C15—O10—H15C	50.1
N11—N5—Fe1	121.89 (9)	C15—O10—H15B	56.0
N1—N7—C7	110.79 (12)	H15C—O10—H15B	105.9
N1—N7—C11	107.95 (11)	O6—C12—O2	134.3 (9)
C7—N7—C11	109.38 (12)	O6—C12—C12^i^	113.8 (10)
N3—N9—C9	110.80 (12)	O2—C12—C12^i^	107.6 (9)
N3—N9—C7	109.02 (12)	O6—C12—H12A	116.5
C9—N9—C7	110.14 (12)	O2—C12—H12A	62.4
N5—N11—C11	111.24 (12)	C12^i^—C12—H12A	110.6
N5—N11—C9	108.82 (11)	O6—C12—H12B	94.6
C11—N11—C9	109.88 (12)	O2—C12—H12B	51.9
H13D—N13—H13E	108.6 (18)	C12^i^—C12—H12B	110.6
H13D—N13—H13F	106.2 (18)	H12A—C12—H12B	109.5
H13E—N13—H13F	112.3 (18)	O6—C12—H12C	14.8
H13D—N13—H13G	110.3 (18)	O2—C12—H12C	144.8
H13E—N13—H13G	109.8 (19)	C12^i^—C12—H12C	107.2
H13F—N13—H13G	109.6 (19)	H12A—C12—H12C	109.5
O1—C1—N1	128.86 (14)	H12B—C12—H12C	109.5
O1—C1—C3^i^	119.45 (13)	H15A—C15—O10	127.8
N1—C1—C3^i^	111.69 (13)	H15A—C15—O4	8.8
O3—C3—N3	128.37 (14)	O10—C15—O4	136.6 (10)
O3—C3—C1^i^	120.94 (14)	H15A—C15—C15^i^	113.7
N3—C3—C1^i^	110.69 (13)	O10—C15—C15^i^	117.4 (11)
O5—C5—N5	127.40 (14)	O4—C15—C15^i^	105.1 (10)
O5—C5—C5^i^	121.93 (9)	H15A—C15—H15C	109.5
N5—C5—C5^i^	110.67 (8)	O10—C15—H15C	64.6
N7—C7—N9	113.67 (12)	O4—C15—H15C	112.1
N7—C7—H3A	108.8	C15^i^—C15—H15C	107.3
N9—C7—H3A	108.8	H15A—C15—H15B	109.5
N7—C7—H3B	108.8	O10—C15—H15B	45.0
N9—C7—H3B	108.8	O4—C15—H15B	115.1
H3A—C7—H3B	107.7	C15^i^—C15—H15B	107.3
N9—C9—N11	113.19 (13)	H15C—C15—H15B	109.5
N9—C9—H4A	108.9	H15A—C15—H15A	0.0
N11—C9—H4A	108.9	O10—C15—H15A	127.8
N9—C9—H4B	108.9	O4—C15—H15A	8.8
N11—C9—H4B	108.9	C15^i^—C15—H15A	113.7
H4A—C9—H4B	107.8	H15C—C15—H15A	109.5
N11—C11—N7	113.98 (12)	H15B—C15—H15A	109.5
N11—C11—H6A	108.8		
			
C1—N1—N7—C7	−146.37 (14)	N9—N3—C3—C1^i^	−168.31 (12)
Fe1—N1—N7—C7	58.47 (15)	Fe1—N3—C3—C1^i^	−19.18 (16)
C1—N1—N7—C11	93.89 (15)	N11—N5—C5—O5	15.8 (2)
Fe1—N1—N7—C11	−61.27 (14)	Fe1—N5—C5—O5	169.60 (13)
C3—N3—N9—C9	−154.44 (13)	N11—N5—C5—C5^i^	−164.79 (14)
Fe1—N3—N9—C9	58.19 (15)	Fe1—N5—C5—C5^i^	−11.0 (2)
C3—N3—N9—C7	84.19 (15)	N1—N7—C7—N9	−65.02 (16)
Fe1—N3—N9—C7	−63.18 (14)	C11—N7—C7—N9	53.87 (16)
C5—N5—N11—C11	−148.26 (13)	N3—N9—C7—N7	67.24 (16)
Fe1—N5—N11—C11	58.90 (15)	C9—N9—C7—N7	−54.54 (16)
C5—N5—N11—C9	90.56 (15)	N3—N9—C9—N11	−66.79 (16)
Fe1—N5—N11—C9	−62.28 (14)	C7—N9—C9—N11	53.92 (16)
N7—N1—C1—O1	11.1 (2)	N5—N11—C9—N9	68.10 (16)
Fe1—N1—C1—O1	167.61 (13)	C11—N11—C9—N9	−53.92 (16)
N7—N1—C1—C3^i^	−168.97 (12)	N5—N11—C11—N7	−66.46 (16)
Fe1—N1—C1—C3^i^	−12.47 (17)	C9—N11—C11—N7	54.10 (16)
N9—N3—C3—O3	11.7 (2)	N1—N7—C11—N11	66.44 (16)
Fe1—N3—C3—O3	160.86 (14)	C7—N7—C11—N11	−54.19 (16)

Symmetry code: (i) −*x*+1, *y*, −*z*+3/2.

### Hydrogen-bond geometry (Å, º)

**Table:**

*D*—H···*A*	*D*—H	H···*A*	*D*···*A*	*D*—H···*A*
O2—H2···O7^ii^	0.84	1.94	2.763 (6)	165
O2—H12*B*···N1	1.00	2.66	3.338 (6)	125
O2—H12*A*···O5^iii^	1.17	2.58	3.356 (6)	122
O4—H4···O7^ii^	0.84	1.96	2.792 (7)	169
O8—H8···O1^iv^	0.81 (3)	1.80 (3)	2.6007 (17)	168 (3)
O10—H15*C*···O5^iii^	1.15	2.39	3.425 (6)	148
N13—H13*D*···O7^v^	0.94 (2)	1.99 (2)	2.913 (2)	166.4 (18)
N13—H13*E*···O3^vi^	0.91 (2)	2.00 (2)	2.9073 (19)	173.2 (19)
N13—H13*E*···N9^vi^	0.91 (2)	2.64 (2)	3.146 (2)	116.5 (16)
N13—H13*F*···O5	0.88 (2)	2.12 (2)	2.9606 (19)	160.7 (19)
N13—H13*F*···N11	0.88 (2)	2.63 (2)	3.252 (2)	128.3 (17)
N13—H13*G*···O1^vii^	0.87 (2)	2.09 (2)	2.9241 (19)	158.8 (19)
N13—H13*G*···O3^ii^	0.87 (2)	2.48 (2)	3.0698 (19)	125.0 (17)
C7—H3*A*···O5^viii^	0.99	2.47	3.4109 (19)	159
C7—H3*B*···O10	0.99	2.43	3.366 (6)	158
C9—H4*A*···O2^vi^	0.99	2.35	3.312 (7)	165
C9—H4*A*···O10^vi^	0.99	2.17	3.132 (5)	165
C11—H6*B*···O1^vii^	0.99	2.31	3.2606 (19)	160
C12—H12*B*···N1	0.98	2.66	3.575 (7)	155
C12—H12*A*···O5^iii^	0.98	2.58	3.448 (7)	147
C13—H13*B*···O5	0.90 (3)	2.67 (3)	3.480 (2)	151 (2)
C13—H13*C*···O8^ix^	0.91 (3)	2.63 (3)	3.416 (3)	146 (2)
C15—H15*C*···O5^iii^	0.98	2.39	3.245 (6)	146

Symmetry codes: (ii) *x*, −*y*+1, *z*−1/2; (iii) *x*, *y*−1, *z*; (iv) *x*, −*y*+1, *z*+1/2; (v) *x*, −*y*+2, *z*−1/2; (vi) −*x*+3/2, *y*+1/2, −*z*+3/2; (vii) −*x*+1, −*y*+1, −*z*+1; (viii) −*x*+3/2, *y*−1/2, −*z*+3/2; (ix) −*x*+3/2, −*y*+3/2, −*z*+2.

## References

[bb1] Blakemore, J. D., Crabtree, R. H. & Brudvig, G. W. (2015). *Chem. Rev.* **115**, 12974–13005. 10.1021/acs.chemrev.5b00122 26151088

[bb2] Boniolo, M., Hossain, M. K., Chernev, P., Suremann, N. F., Heizmann, P. A., Lyvik, A. S. L., Beyer, P., Haumann, M., Huang, P., Salhi, N., Cheah, M. H., Shylin, S. I., Lundberg, M., Thapper, A. & Messinger, J. (2022). *Inorg. Chem.* **61**, 9104–9118. 10.1021/acs.inorgchem.2c00631 PMC9214691 35658429

[bb3] Clark, R. C. & Reid, J. S. (1995). *Acta Cryst.* A**51**, 887–897.

[bb4] *CrystalMaker* (2017). *CrystalMaker*. CrystalMaker Software, Bicester, England.

[bb5] Gil-Sepulcre, M. & Llobet, A. (2022). *Nat. Catal.* **5**, 79–82.

[bb6] Groom, C. R., Bruno, I. J., Lightfoot, M. P. & Ward, S. C. (2016). *Acta Cryst.* B**72**, 171–179. 10.1107/S2052520616003954 PMC4822653 27048719

[bb7] Plutenko, M. O., Shova, S., Pavlenko, V. A., Golenya, I. A. & Fritsky, I. O. (2023). *Acta Cryst.* E**79**, 1059–1062. 10.1107/S2056989023008587 PMC10626965 37936846

[bb8] Rigaku OD (2015). *CrysAlis PRO*. Rigaku Oxford Diffraction, Yarnton, England.

[bb9] Sheldrick, G. M. (2015*a*). *Acta Cryst.* A**71**, 3–8.

[bb10] Sheldrick, G. M. (2015*b*). *Acta Cryst.* C**71**, 3–8.

[bb11] Shylin, S. I., Pavliuk, M. V., D’Amario, L., Fritsky, I. O. & Berggren, G. (2019*a*). *Faraday Discuss.* **215**, 162–174. 10.1039/c8fd00167g PMC6677028 30951052

[bb12] Shylin, S. I., Pavliuk, M. V., D’Amario, L., Mamedov, F., Sá, J., Berggren, G. & Fritsky, I. O. (2019*b*). *Chem. Commun.* **55**, 3335–3338. 10.1039/c9cc00229d 30801592

[bb13] Shylin, S. I., Pogrebetsky, J. L., Husak, A. O., Bykov, D., Mokhir, A., Hampel, F., Shova, S., Ozarowski, A., Gumienna-Kontecka, E. & Fritsky, I. O. (2021). *Chem. Commun.* **57**, 11060–11063. 10.1039/d1cc04870h 34610631

[bb14] Tomyn, S., Shylin, S. I., Bykov, D., Ksenofontov, V., Gumienna-Kontecka, E., Bon, V. & Fritsky, I. O. (2017). *Nat. Commun.* **8**, 14099. 10.1038/ncomms14099 PMC5253674 28102364

[bb15] Westrip, S. P. (2010). *J. Appl. Cryst.* **43**, 920–925.

[bb16] Xu, Y., Hu, Z.-B., Wu, L.-N., Li, M.-X., Wang, Z.-X. & Song, Y. (2020*a*). *Polyhedron*, **175**, 114243.

[bb17] Xu, Y., Wu, L.-N., Li, M.-X., Shi, F.-N. & Wang, Z.-X. (2020*b*). *Inorg. Chem. Commun.* **117**, 107950.

